# NEFAs Influence the Inflammatory and Insulin Signaling Pathways Through TLR4 in Primary Calf Hepatocytes *in vitro*

**DOI:** 10.3389/fvets.2021.755505

**Published:** 2021-12-13

**Authors:** Qinghua Deng, Liyin Du, Yuming Zhang, Guowen Liu

**Affiliations:** ^1^College of Animal Science and Technology, Inner Mongolia Minzu University, Tongliao, China; ^2^Inner Mongolia Minzu University Key Laboratory for Prevention and Control of Herbivorous Livestock Perinatal Diseases, Tongliao, China; ^3^College of Veterinary Medicine, Jilin University, Changchun, China

**Keywords:** NEFAs, insulin resistance, NF-κB signaling pathway, lipid metabolism, bovine hepatocytes

## Abstract

Transition dairy cows are often in a state of negative energy balance because of decreased dry matter intake and increased energy requirements, initiating lipid mobilization and leading to high serum β-hydroxybutyrate (BHBA) and non-esterified fatty acid (NEFAs) levels, which can induce ketosis and fatty liver in dairy cows. Inflammation and insulin resistance are also common diseases in the perinatal period of dairy cows. What is the relationship between negative energy balance, insulin resistance and inflammation in dairy cows? To study the role of non-esterified fatty acids in the nuclear factor kappa beta (NF-κB) inflammatory and insulin signaling pathways through Toll-like receptor 4 (TLR4), we cultured primary calf hepatocytes and added different concentrations of NEFAs to assess the mRNA and protein levels of inflammatory and insulin signaling pathways. Our experiments indicated that NEFAs could activate the NF-κB inflammatory signaling pathway and influence insulin resistance through TLR4. However, an inhibitor of TLR4 alleviated the inhibitory effects of NEFAs on the insulin pathway. In conclusion, all of these results indicate that high-dose NEFAs (2.4 mM) can activate the TLR4/NF-κB inflammatory signaling pathway and reduce the sensitivity of the insulin pathway through the TLR4/PI3K/AKT metabolic axis.

## Introduction

Ketosis and fatty liver, common nutritional, and metabolic diseases in perinatal dairy cows, are characterized by negative energy balance (NEB), often accompanied by insulin resistance and inflammation (1–3). Under NEB conditions, dairy cows mobilize their body fat to produce a large amount of non-esterified fatty acids (NEFAs) into the blood. Compared with cows with medium body condition scores and low body condition scores, cows with high body condition scores at the late lactation stage may have accumulated more hepatic triacylglycerol and lower antioxidant potential due to greater insulin resistance (4). Studies in obese or high-fat diet-fed mice have demonstrated that NEFAs are closely related to inflammation and insulin resistance and act as signaling molecules that can directly regulate lipid metabolism in hepatocytes (5).

Insulin is a regulator of fat production (6). Insulin resistance is a serious disease that can lead to complications such as hyperglycemia and hyperlipidemia throughout the body, including the liver, and other tissues. NEFAs are increased in fatty liver and play an important role in insulin resistance, and it is clear that NEFAs can induce insulin resistance (7). However, the mechanisms by which NEFAs induce insulin resistance and inflammatory are not clear.

TLRs are a class of pathogen recognition receptors that activate the nuclear factor kappa beta (NF-κB) signaling pathway and promote the transcription of proinflammatory factors (8). Inflammatory factors can influence the transmission of the insulin signaling pathway downstream by different pathways to induce the phosphorylation of insulin receptor substrates at serine residues, which can reduce the physiological function of insulin and result in insulin resistance (9). Recent studies indicate that cows with high body condition scores exhibit insulin resistance and a proinflammatory state in the liver and that TLR4 plays an important role in insulin resistance (10). It is well-established that hepatocyte-derived cytokines and/or chemokines, such as tumor necrosis factor-alpha (TNF-α), interleukins (ILs) and macrophage chemoattractant proteins, are involved in the onset and progression of insulin resistance and fatty liver. Activation of inflammatory pathways in obese subjects, such as NF-κB, interferes with insulin signaling (11–13). In the tissue, skeletal muscle, pancreas and liver, insulin resistance is induced by the inflammatory response (14, 15). Furthermore, studies have indicated that NEFAs can activate TLR4 *in vitro* and *in vivo* (16). However, no studies on the relationship between NEFAs, TLR4/NF-κB and the insulin resistance signaling pathway, nor how NEFAs cause insulin resistance in primary calf hepatocytes, have been carried out.

In this study, we added NEFAs to calf primary hepatocytes *in vitro* to detect the effects of NEFAs on inflammatory and insulin signaling pathways and illuminate whether NEFAs mediate calf primary hepatocyte inflammation and insulin resistance through TLR4. Calf primary liver cell isolation and culture has been very popular as an *in vitro* experimental model applied to molecular biology experiments (17–20). Therefore, in this study, we used experimental models to study the insulin and inflammatory pathways in cows *in vitro*.

## Materials and Methods

### Reagents

NEFAs were prepared as follows: 4.35 mM oleic acid, 0.49 mM linoleic acid, 3.19 mM palmitic acid, 1.44 mM stearic acid, 0.53 mM palmitic acid, and 113 mL of 0.1 mol/L KOH were heated to 60°C to fully dissolve the above five acids, after which 7.5 mL of 1 M HCl preheated to 60°C was added. After complete mixing, 67.8 mL of five-fold distilled water preheated to 60°C was added, producing a storage solution at 53.4 mM, which was refrigerated at −20°C (19, 21, 22).

TAK-242 was purchased from MedChem-Express (NJ, USA). Anti-β-actin antibody was purchased from Santa Cruz Biotechnology, Inc. (CA, USA). Insulin and DMSO were purchased from Sigma-Aldrich (MO, USA). Fetal bovine serum, collagenase IV, and RPMI-1640 medium were purchased from Gibco (NY, USA). Bovine serum albumin V and reverse transcription PCR and real-time PCR kits were provided by Roche (Basel, Switzerland). IL-6 and TNFα ELISA kits were purchased from R&D Systems. Antibodies against TLR4, p-P65, P65, p-GSK-3β, p-AKT, and AKT were purchased from Cell Signaling Technology (MA, USA). Antibodies against IRS1 and GSK-3β were purchased from Millipore (Massachusetts, USA). Antibodies against PI3K and GLUT4 were purchased from Abcam (Cambridge, England).

### Methods

#### Cell Isolation and Culture

The study protocol was approved by the Ethics Committee on the Use and Care of Animals at Inner Mongolia University for Nationalities (Tongliao, China). The caudate lobe was obtained from a newborn fasted female Holstein calf (1 day old, BW: 30–40 kg) anesthetized with thiamylal sodium under sterile conditions by surgical liver excision. Hepatocytes were isolated with a modified two-step collagenase perfusion protocol (23). The liver was perfused with perfusion solution to wash away the blood until the perfusion solution became clear. The liver was then perfused with a collagenase IV solution to digest the liver tissue until the liquid became opaque. The liver capsule was cut off after digestion. We used 100 mL of precooled RPMI-1640 culture medium containing 0.2% bovine serum albumin to terminate the digestion (basic RPMI-1640 culture medium was prepared according to the manufacturer's protocol and consisted of 26 mM NaHCO_3_, 10 mM HEPES, and 20 mM NaCl with an adjusted pH of 7.2). The liver capsule, blood vessels, fat, and other parts of the liver caudate lobe were then discarded. The hepatocyte suspension was sequentially filtered with 100-mesh (150 μm) and 200-mesh (75 μm) cell sieves. The cell density was adjusted to 2×10^6^ cells/mL with adherent RPMI-1640 culture medium. Hepatocytes were seeded in a 6-well-tissue culture plate (2 mL per well) and incubated at 37°C in 5% CO_2_. Every 24 h, the medium was replaced with fresh medium, and the hepatocyte shape and growth conditions were observed daily.

#### Hepatocyte Treatment

Before the addition stimuli, the hepatocytes were cultured in serum-free RPMI-1640 medium for 24 h. Then, we exchanged the RPMI-1640 medium with RPMI-1640 medium containing 2% BSA when we added the NEFAs to the hepatocytes. The hepatocytes were divided into four or seven groups in different experiments. The NEFAs groups were as follows: control (0 mM NEFAs), low-concentration group (0.6 mM NEFAs), middle-concentration group (1.2 mM NEFAs) and high-concentration group (2.4 mM NEFAs). The second group was as follows: a TLR4 inhibitor (TAK-242, 1μM) group, DMSO group, 2.4 mM NEFAs + TAK-242 group and 2.4 mM NEFAs group. The third group was as follows: control (0 mM NEFAs), low-concentration group (0.6 mM NEFAs), middle-concentration group (1.2 mM NEFAs) and high-concentration group (2.4 mM NEFAs), a TLR4 inhibitor (TAK-242) group, DMSO group, 2.4 mM NEFAs + TAK-242 group. On the basis of the third group, insulin (100 nM) was added to the fourth group. Each treatment was replicated 6 times. In this paper, replicates refer to biological replicates and (*n*) refer to the number of independent values. After 9 h, the total RNA and protein were extracted (22). TAK-242 was added to a concentration of 1 μM, and NEFAs at a high concentration were added 1 h later. In the insulin addition group, 100 nM insulin was added 0.5 h before collection. The collected protein, RNA and cell supernatants were stored at −80°C.

#### Quantitative Real-Time PCR

Total RNA was extracted from the cells using TRIzol according to the manufacturer's instructions (TaKaRa Biotechnology Co., Ltd., Tokyo, Japan). According to the manufacturer's instructions, the RNA was reverse transcribed into cDNA using a reverse transcription kit (TaKaRa Biotechnology Co., Ltd., Tokyo, Japan). All primers were synthesized by GENEWIZ (GENEWIZ Biotech Co., Ltd., Suzhou, China). mRNA expression levels were evaluated by quantitative real-time PCR analysis using the SYBR Green QuantiTect RT-PCR Kit (Roche, Basel, Switzerland). The mRNA expression levels were normalized to the mRNA levels of the housekeeping gene β-actin. Real-time PCR was conducted under the following conditions: initial denaturation at 95°C for 3 min and 45 amplification cycles (denaturation at 95°C for 15 s, annealing at 60°C for 1 min). Relative gene expression was calculated by the 2^−ΔΔCt^ method and normalized to the level of β-actin (24).

#### Protein Extraction and Western Blot Analysis

Total protein was extracted from hepatocytes using lysis buffer (Pierce, Rockford, IL, USA), and the protein concentration was measured using a BCA protein assay kit (Pierce, Rockford, IL, USA). Prepared proteins were subjected to standard SDS-PAGE with 10% (w/v) polyacrylamide gels and electrotransferred onto PVDF membranes (Roche, Basel, Switzerland) using a wet blotting apparatus. Then, the membranes were incubated in blocking solution (3% bovine serum albumin V in Tris-HCl buffer) for 4 h at room temperature and incubated overnight at 4°C with antibodies against TLR4, p-P65, P65, p-IRS1, IRS1, p-IRS2, IRS2, PI3K, p-AKT, AKT, p-GSK-3β, GSK-3β, GLUT4, and β-actin. The membranes were then incubated while shaking three times for 5 min each with TBS containing 0.1% Tween 20, incubated with the appropriate peroxidase-conjugated secondary antibodies (Protein Technology, Chicago, IL, USA) for 45 min while shaking at room temperature and washed four times for 5 min each. The resulting bands were detected with an ECL kit (Millipore, Boston, Massachusetts, USA). The relative expression levels of the proteins were normalized to the β-actin level (25).

#### Analysis of Inflammatory Factors in Cell Culture Supernatants

The cell culture supernatants were slowly dissolved at room temperature, and the ELISA kits were incubated at room temperature for at least half an hour. The supernatants were then centrifuged for 10 min at 300 × g, and the supernatants were collected in a new centrifuge tube. Then, we detected the levels of the inflammatory factors TNFα and IL-6 according to the instructions (22).

#### Cell Immunofluorescence

Preparation of glass coverslips: After cleaning the immunofluorescence-specific glass coverslips, we added them to 75% ethyl alcohol containing 1% HCl for incubation overnight. Then, we soaked the coverslips in double distilled water for 2 h, and after drying, we placed them in acid for 36 h, removed them and washed them with running water 3 times. The coverslips were then soaked with absolute ethyl alcohol overnight and finally baked at 180°C for 3 h (26).

(1) The glass coverslips were placed into a 24-well-cell culture plate, and 500 μL of hepatocytes at a density of 5×10^5^ cells/mL were added to each well.
(2) For normal cell culture, the cells were washed 3 times with serum-free medium for 5 min each and then washed with 0.01% PBS.
(3) We then fixed the cells with 4% paraformaldehyde or 10% formalin for 20 min (no more than 30 min) and washed them 3 times with 0.01% PBS for 5 min each.
(4) After EDTA Na_2_ antigen repair (95°C for 5 min), when the temperature had decreased to room temperature, the cells were washed 3 times with 0.01% PBS for 5 min each.
(5) The cells were lysed with 0.1% Triton-100 (300 mL) for 10 min and then washed 3 times with 0.01% PBS for 5 min each.
(6) After the cells were incubated with anti-P65 antibody diluted in goat serum at 4°C overnight, they were washed 3 times with 0.01% PBS for 5 min each.
(7) We then incubated the cells with the appropriate peroxidase-conjugated secondary antibody (Protein Technology, Chicago, IL, USA) for 30 min while avoiding light and then washed them 3 times with 0.01% PBS for 5 min each.
(8) DAPI nuclear dye was added and incubated while avoiding light for 7 min.
(9) Finally, confocal fluorescence was used to observe the cells.

#### Statistical Analyses

All experiments were repeated at least 3 times, and data were analyzed using GraphPad Prism 5 (Graph-Pad InStat Software) and SPSS (Statistical Package for the Social Sciences) 13.0 software (SPSS Inc., Chicago, IL, USA) to analyze the experimental data. All data were tested for normality and homogeneity of variance. For data with Gaussian distribution, unpaired 2-sided Student *t*-test and oneway ANOVA with a Bonferroni post-test were used to compare differences between 2 groups or more than 2 nonparametric statistical analyses was performed using the Mann Whitney *U*-test and Kruskal-Wallis test with Dunn's post-hoc test for 2 groups or more than 2 groups, respectively. Data are expressed as means ± standard error. A significant difference was defined as *P* < 0.05, and *P* < 0.01 was considered highly significant.

## Results

### NEFAs Affected the Expression of TLR4 and Activated the Inflammatory Pathway in Primary Calf Hepatocytes

**Figure 1:**
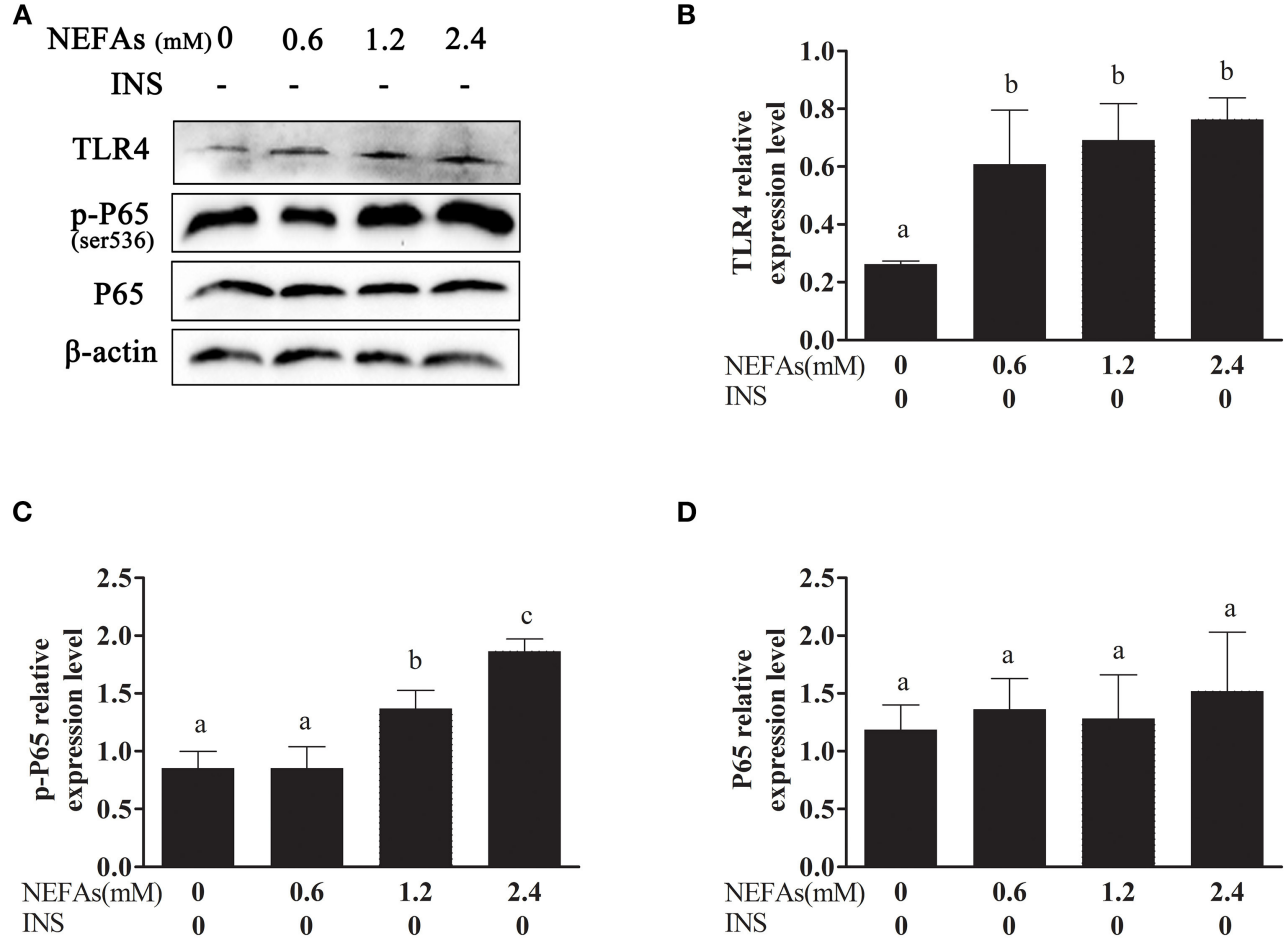
Effects of NEFAs on TLR4 and the inflammatory pathway. Protein expression levels after 9 h incubation with different concentration of NEFAs (0 mM, 0.6, 1.2, and 2.4 mM) were determined by western blotting. **(A)** Bands of TLR4, p-P65, and P65. **(B–D)** Relative expression levels of TLR4 **(B)**, p-P65 **(C)**, and P65 **(D)**. The relative expression levels are expressed as the optical density ratio compared against β-actin levels. Data are presented as the mean ± SE (*n* = 3), and different letters indicate a statistically significant difference; *p* < 0.05.

As shown in Figure 1, with increasing NEFAs concentration, the protein expression of TRL4 tended to increase and peaked in the high-concentration group (*p* < 0.05). The protein expression levels of p-P65 in the medium- and high-concentration groups were significantly higher than those in the control and low-concentration groups (*p* < 0.05), but the protein expression of P65 was not significantly different among the four groups (*p* > 0.05).

### Effects of NEFAs on the Expression of Related Proteins in the Insulin Signaling Pathway

**Figure 2:**
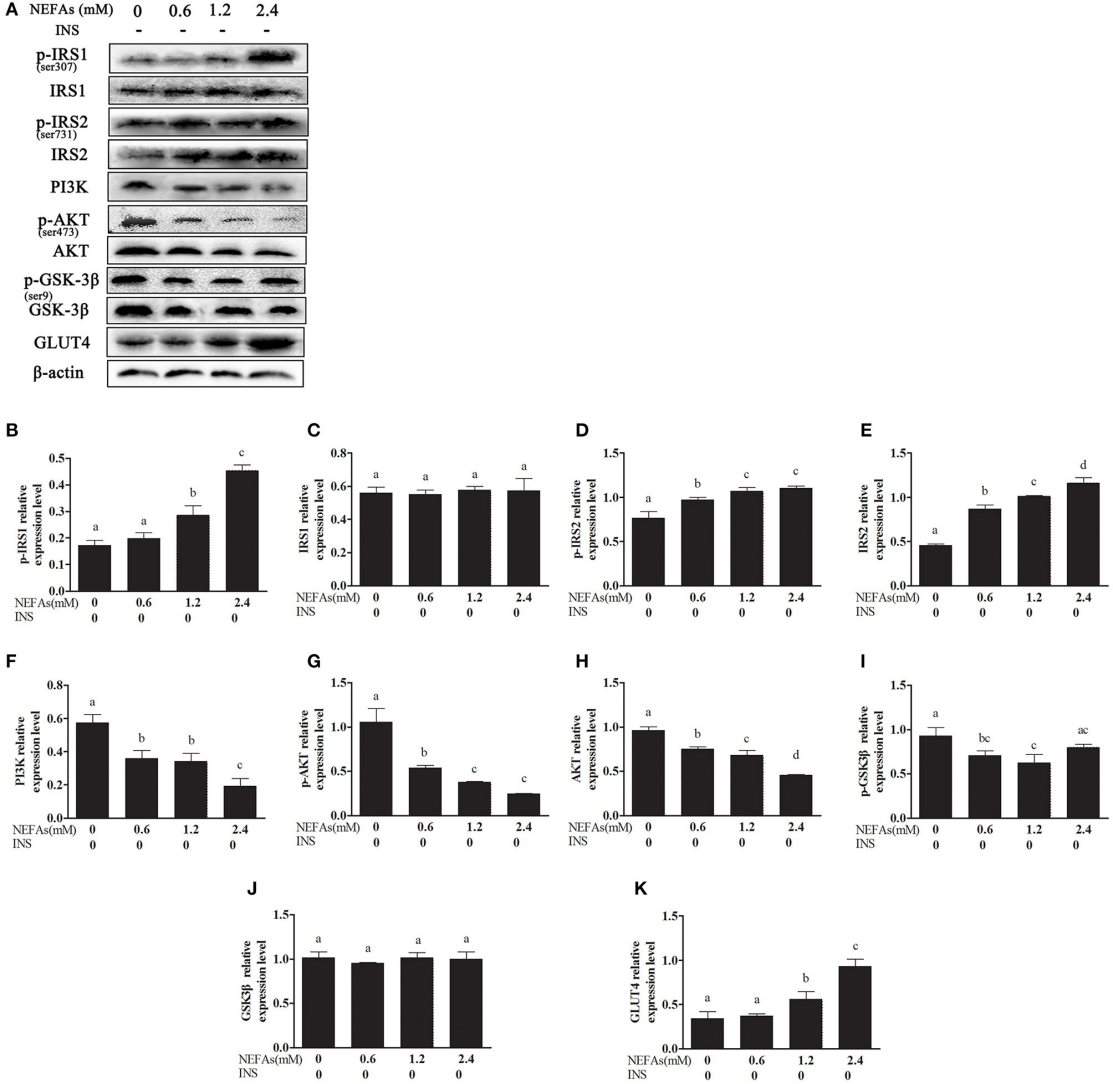
Effects of NEFAs on the expression of related proteins in the insulin signaling pathway. Protein expression levels after 9 h incubation with different concentration of NEFAs (0, 0.6, 1.2, and 2.4 mM) were determined by western blotting. **(A)** Bands of p-IRS1, IRS1, p-IRS2, IRS2, PI3K, p-AKT, AKT, p-GSK-3β, GSK-3β, and GLT4. **(B–K)** Relative expression levels of p-IRS1 **(B)**, IRS1 **(C)**, p-IRS2 **(D)**, IRS2 **(E)**, PI3K **(F)**, p-AKT **(G)**, AKT **(H)**, p-GSK-3β **(I)**, GSK-3β **(J)**, and GLT4 **(K)**. The relative expression levels are expressed as the optical density ratio compared against β-actin levels. Data are presented as the mean ± SE (*n* = 3), and different letters indicate a statistically significant difference; *p* < 0.05.

With increasing NEFAs concentrations, the protein levels of p-IRS1, p-IRS2, IRS2, and GLUT4 increased (*p* < 0.05) and peaked in the high-concentration group. In contrast, the expression levels of PI3K, p-AKT, AKT, and p-GSK3β were significantly decreased (*p* < 0.05) and were lowest in the high-concentration group. However, the expression levels of IRS1 and GSK-3β were not significantly changed with NEFAs application (*p* > 0.05; Figure 2).

### NEFAs Affected the NF-κB Inflammatory Pathway Through the TLR4 Protein

**Figure 3:**
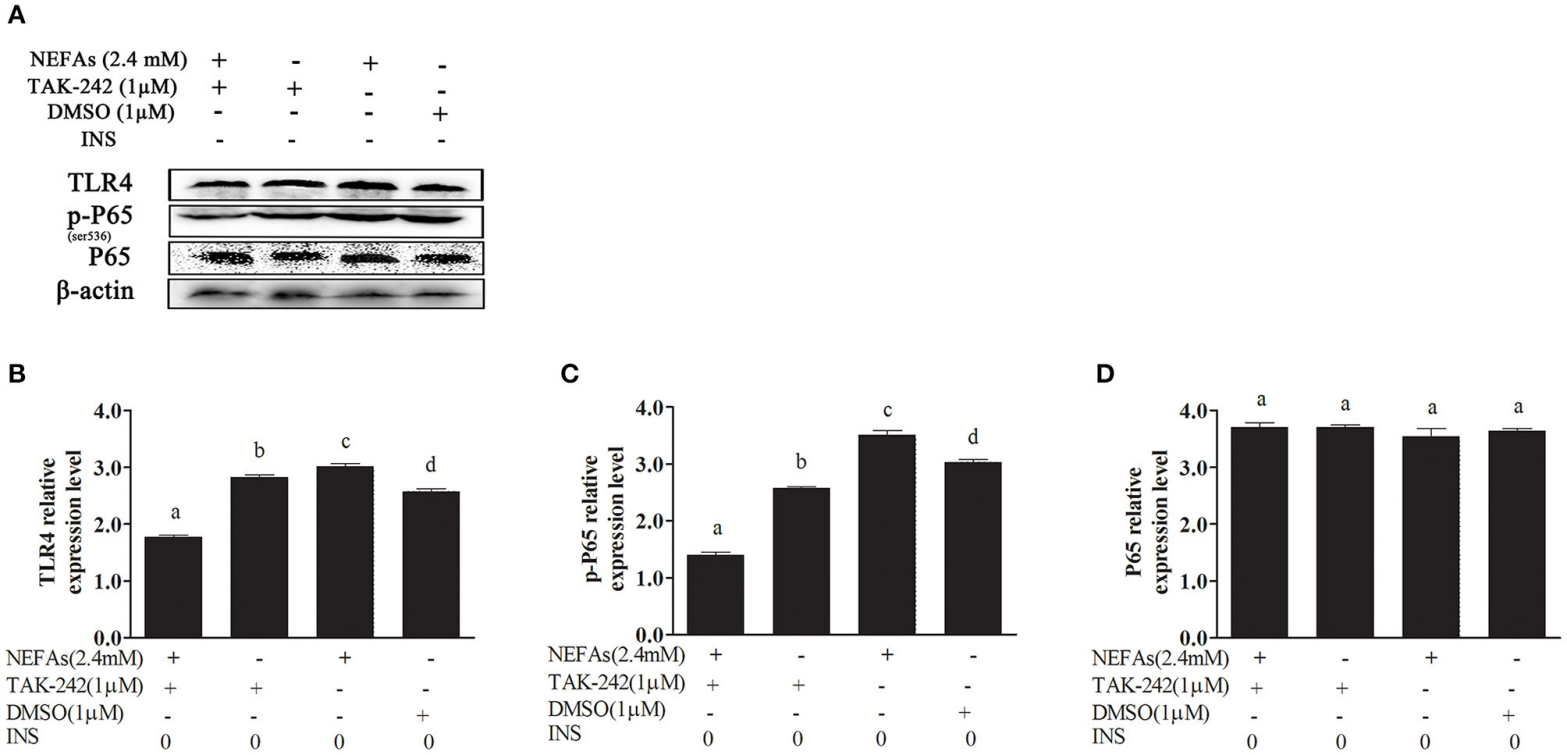
NEFAs affected the NF-κB inflammatory pathway through the TLR4 protein. Protein expression levels were determined by western blotting. Hepatocytes were treated with TLR4 inhibitor (TAK-242, 1 μM), DMSO (1 μM) and NEFAs (2.4 mM) for 9 h. **(A)** Bands of TR4, p-P65, and P65. **(B–D)** Relative expression levels of TLR4 **(B)**, p-P65 **(C)**, and P65 **(D)**. The relative expression levels are expressed as the optical density ratio compared against β-actin levels. Data are presented as the mean ± SE (*n* = 3), and different letters indicate a statistically significant difference; *p* < 0.05.

We added TAK-242, an inhibitor of TLR4, and detected its effect on the inflammatory pathway. Hepatocytes were divided into four groups: the NEFAs + TAK-242 group, TAK-242 group, NEFAs (2.4 mM) group, and DMSO group. The TLR4 and p-P65 protein levels in the NEFAs group were higher than those in the DMSO group (*p* < 0.05); however, after the addition of the inhibitor, namely, in the NEFAs + TAK-242 group, the protein level of TLR4 was significantly lower than that in the NEFAs group (*p* < 0.05). However, the level of P65 showed no obvious change (*p* > 0.05; Figure 3).

### Analysis of the Effects of NEFAs on the NF-κB Inflammatory Pathways Through TLR4 by Cell Immunofluorescence

**Figure 4:**
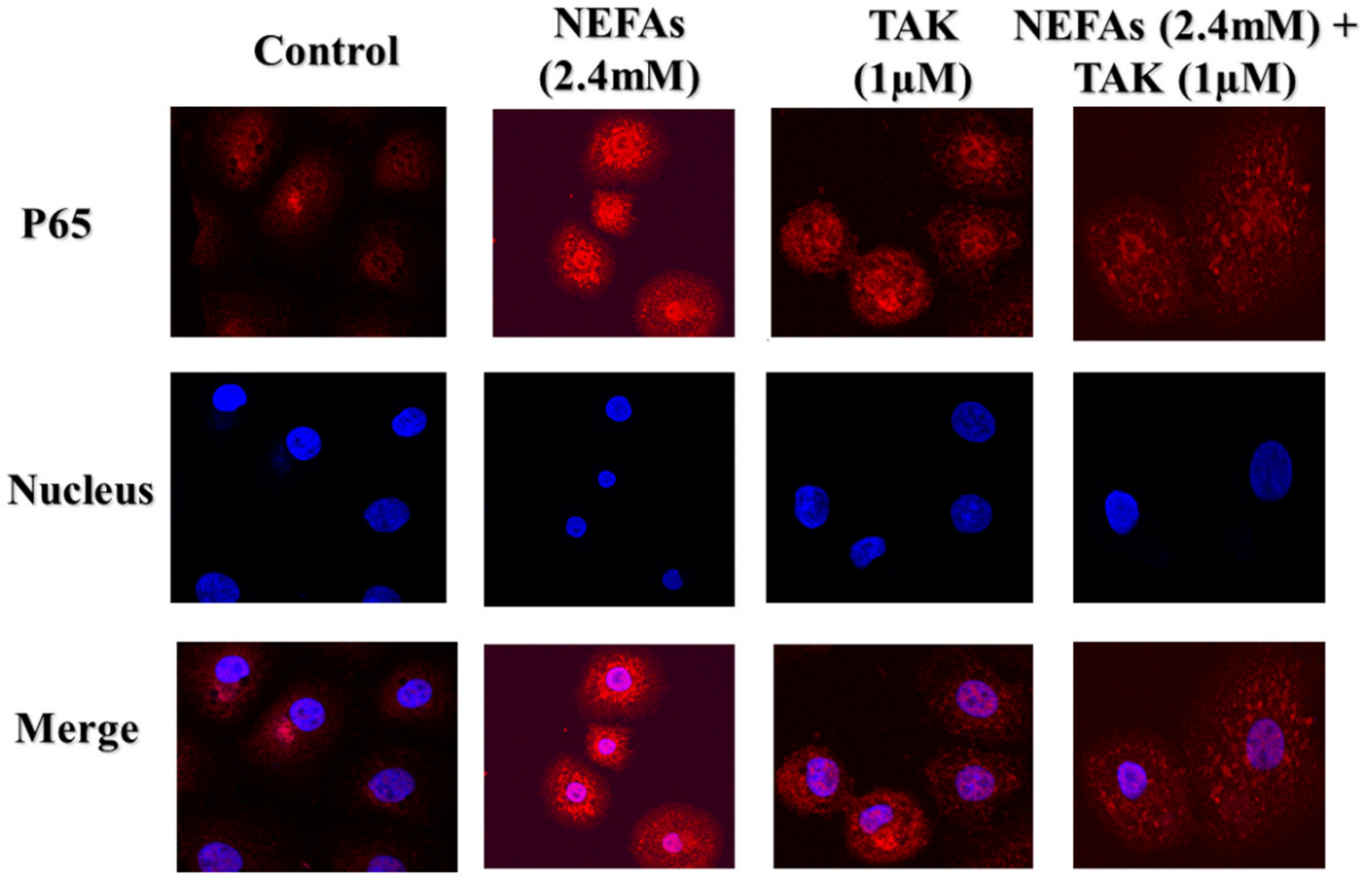
Representative images (original magnification 800×, bar scale: 100 μm, *n* = 3) of immunofluorescence analysis of P65 in primary calf hepatocytes. Hepatocytes were treated with TLR4 inhibitor (TAK-242, 1μM), DMSO (1μM), and NEFAs (2.4 mM) for 9 h.

To further test whether NEFAs affect inflammatory pathways through TLR4, we used immunofluorescence to detect the effects of NEFAs and TAK-242 on the nuclear transfer of P65. The expression of P65 in the nucleus and cytoplasm was obviously higher in the NEFAs groups than in the control group. The expression of P65 in the nucleus and cytoplasm was lower in the NEFAs + TAK-242 group than in the high-concentration group (Figure 4).

### NEFAs Affected Inflammatory Factors Through TLR4 at the MRNA Level

**Figure 5:**
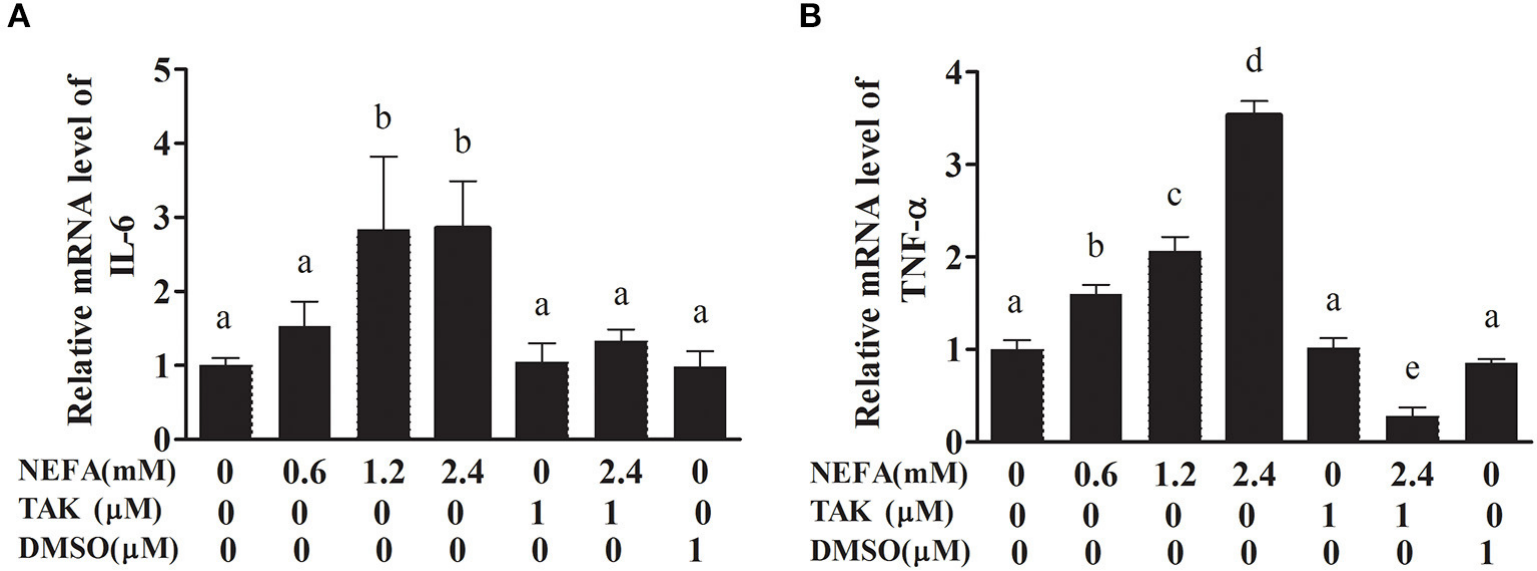
Effects of NEFAs and TAK-242 on the mRNA expression levels of IL-6 and TNFα. Hepatocytes were treated with NEFAs (2.4 mM), TAK-242 (1 μM), and DMSO (1 μM) for 9 h. **(A)** Relative mRNA level of IL-6. **(B)** Relative mRNA level of TNF-α. Values are the means ± SEs (*n* = 6), and different letters indicate a statistically significant difference; *p* < 0.05.

We analyzed the effects of NEFAs on the mRNA levels of the inflammatory factors IL-6 and TNFα. The primer sequences specific for IL-6 and TNFα are listed in Table 1. The IL-6 mRNA level showed no obvious difference between the low-concentration group and the control group (*p* > 0.05), but the medium- and high-concentration groups contained IL-6 mRNA levels clearly higher than those in the control group (*p* < 0.05). However, the addition of TAK-242 obviously decreased the mRNA level of IL-6 compared to that in the NEFAs group (*p* < 0.05). There was no difference in IL-6 mRNA levels between the TAK-242, DMSO and control groups (*p* > 0.05; Figure 5A). The mRNA level of TNFα increased with increasing NEFAs concentration and was highest in the high-concentration group (*p* < 0.05). Furthermore, the mRNA level of TNFα was lower in the TAK-242 + NEFAs group than in the high-concentration group and control group (*p* < 0.05), but there was no difference in TNFα mRNA levels between the TAK-242, DMSO, and control groups (*p* > 0.05; Figure 5B).

**Table 1:** Primers used for qRT-PCR.

**Primers used for PCR (5^′^-3^′^)**	
β-actin	F: GCT AAC AGT CCG CCT AGA AGC A
	R: GTC ATC ACC ATC GGC AAT GAG
IL-6	F: AAC GAG TGG GTA AAG AAC GC
	R: CTG ACC AGA GGA GGG AAT GC
TNF-α	F: CTG CCG GAC TAC CTG GAC TAT
	R: CCT CAC TTC CCT ACA TCC CTA A
G6-Pase	F: AGC AAG TGG TTC CCG TTT C
	R: ACC CAG GCG AGG CAG TA
PEPCK	F: AAG TAC CTT GAG GAG CAA GTG AA
	R: GGT GCG TTG TAT GGA TTG GA

### Effect of NEFAs on Inflammatory Cytokines in the Cell Supernatant

**Figure 6:**
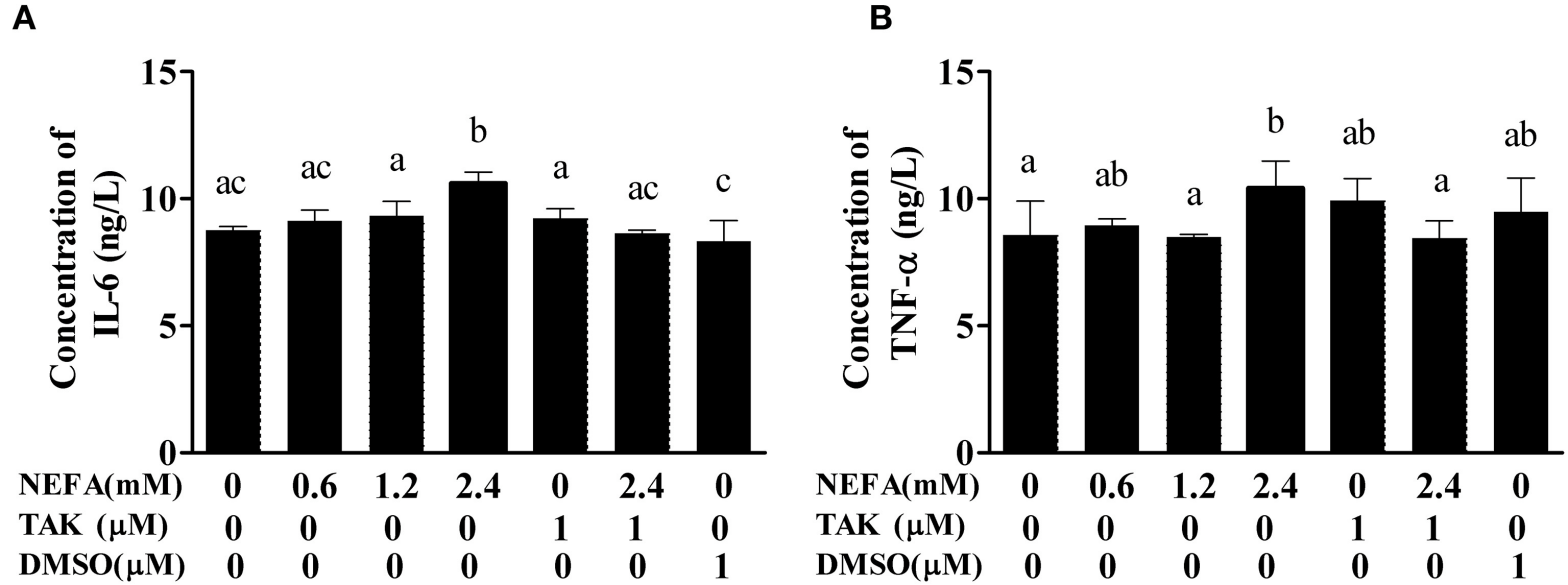
Effects of NEFAs on IL-6 and TNFα in the culture supernatant. Hepatocytes were treated with NEFAs (2.4 mM), TAK-242 (1 μM), and DMSO (1 μM) for 9 h. **(A)** Concentration of IL-6. **(B)** Concentration of TNFα. Values are the means ± SEs (*n* = 6), and different letters indicate statistically significant differences; *p* < 0.05.

To verify that NEFAs influence inflammatory pathways although TLR4, we detected the IL-6 and TNFα levels in the cell supernatant by ELISA. The IL-6 and TNFα levels were obviously increased in the high-concentration group (*p* < 0.05) and lower in the TAK-242 + NEFA group than in the high-concentration group (*p* < 0.05). However, there were no differences between the low- and medium-concentration groups and the control group (*p* > 0.05; Figure 6).

### NEFAs Affect the Insulin Pathway Through TLR4 at the Protein Level

**Figure 7:**
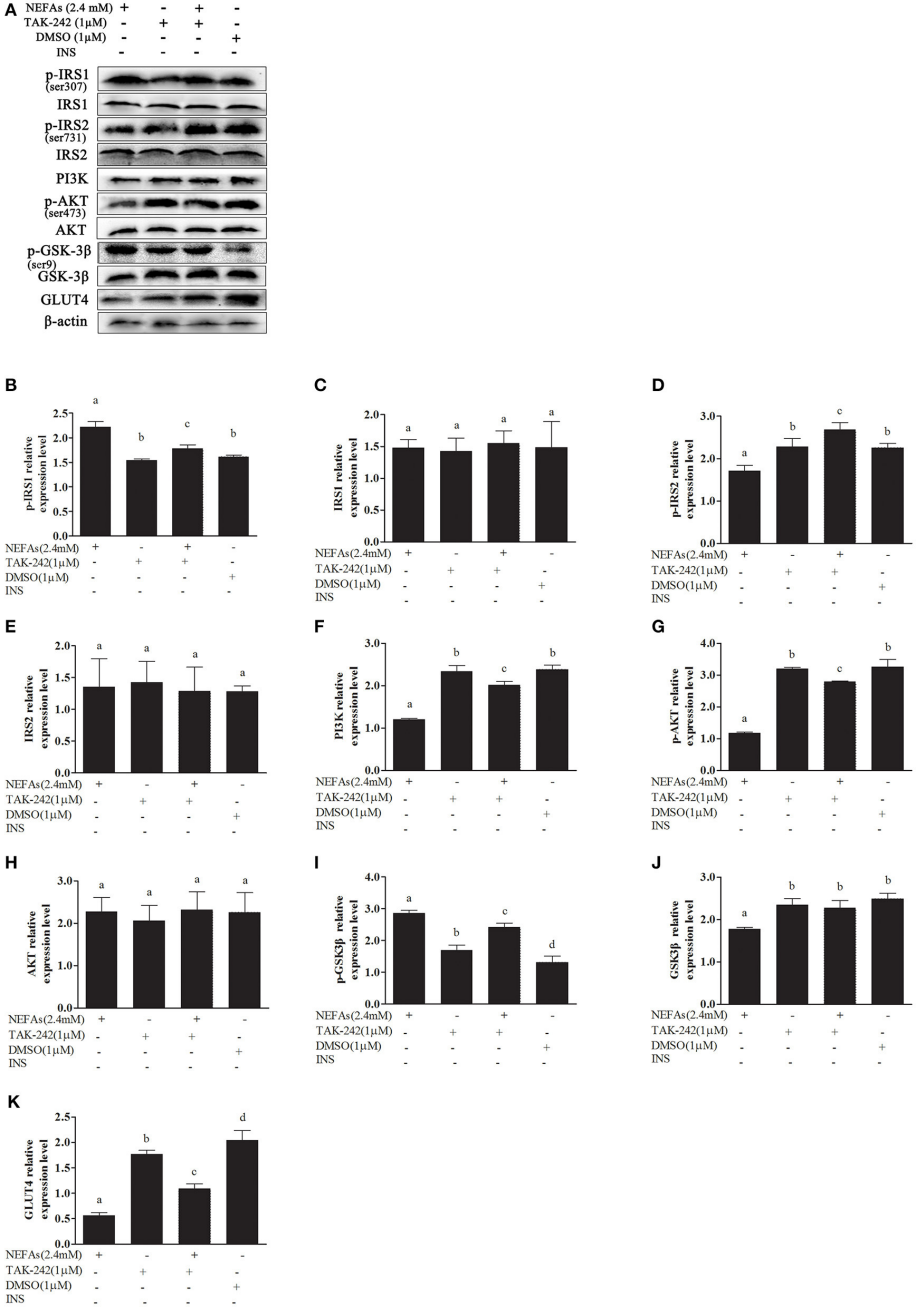
Effects of NEFAs and TAK-242 on key proteins in the insulin signaling pathway. Hepatocytes were treated with NEFAs (2.4 mM), TAK-242 (1 μM), and DMSO (1 μM) for 9 h. Protein expression levels were determined by western blotting. **(A)** Bands of p-IRS1, IRS1, p-IRS2, IRS2, PI3K, p-AKT, AKT, p-GSK-3β, GSK-3β, and GLT4. **(B–K)** Relative expression levels of p-IRS1 **(B)**, IRS1 **(C)**, p-IRS2 **(D)**, IRS2 **(E)**, PI3K **(F)**, p-AKT **(G)**, AKT **(H)**, p-GSK-3β **(I)**, GSK-3β **(J)**, and GLT4 **(K)**. The relative expression levels are expressed as the optical density ratio compared against β-actin levels. Data are presented as the mean ± SE (*n* = 3), and different letters indicate a statistically significant difference; *p* < 0.05.

In the NEFAs group, the phosphorylation levels of GSK-3β and IRS1 were higher (*p* < 0.05), and the protein levels of p-AKT, p-IRS2, GLUT4, and PI3K were significantly lower than those in the DMSO group (*p* < 0.05). There were no significant differences in IRS1, IRS2, or AKT levels between the groups (*p* > 0.05). The protein level of GSK-3β in the NEFAs group was lower than that in the DMSO group (*p* < 0.05), but there was no difference between the other groups (*p* > 0.05). After the addition of the TLR4 inhibitor, the levels of phosphorylated IRS1 and GSK-3β in the NEFAs + TAK-242 group were lower than those in the NEFA group; however, the protein levels of p-AKT, p-IRS2, GLUT4, and PI3K were significantly higher than those in the NEFAs group (*p* < 0.05). There was no difference in protein levels between the TAK-242 and DMSO groups (*p* > 0.05), except for p-GSK-3β and GLUT4 (Figure 7).

**Figure 8:**
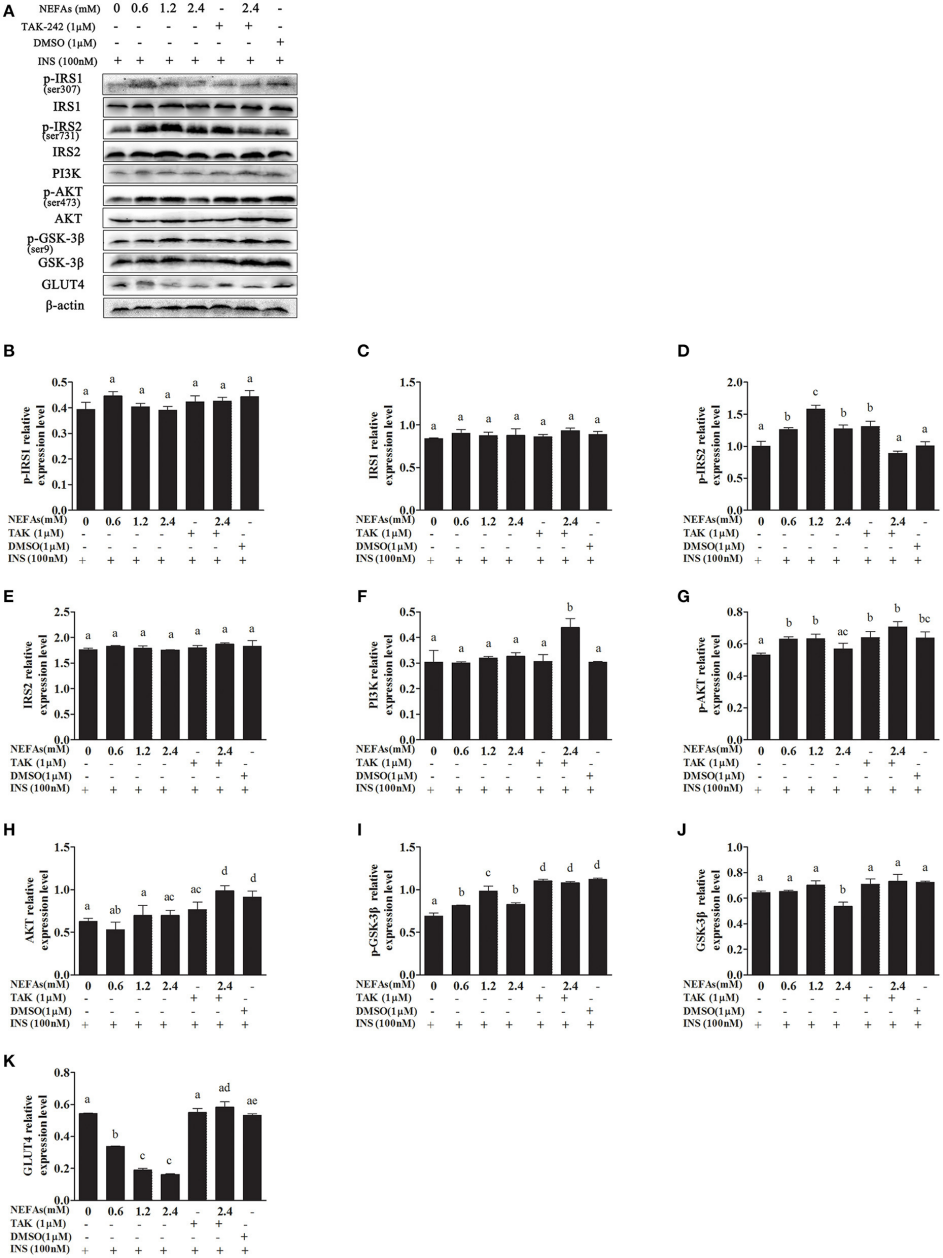
Effects of NEFAs, TAK-242, and INS on key proteins in the insulin signaling pathway. Hepatocytes were treated with NEFAs (2.4 mM), TAK-242 (1 μM), and DMSO (1 μM) for 9 h and insulin (INS, 100 nM) for 1 h. Protein expression levels were determined by western blotting. **(A)** Bands of p-IRS1, IRS1, p-IRS2, IRS2, PI3K, p-AKT, AKT, p-GSK-3β, GSK-3β, and GLT4. **(B–K)** Relative expression levels of p-IRS1 **(B)**, IRS1 **(C)**, p-IRS2 **(D)**, IRS2 **(E)**, PI3K **(F)**, p-AKT **(G)**, AKT **(H)**, p-GSK-3β **(I)**, GSK-3β **(J)**, and GLT4 **(K)**. The relative expression levels are expressed as the optical density ratio compared against β-actin levels. Data are presented as the mean ± SE (*n* = 3), and different letters indicate a statistically significant difference; *p* < 0.05.

We simultaneously added insulin to some cells as a control and found that the expression of p-IRS1 and IRS1 was not different between the groups (*p* > 0.05). The protein level of p-IRS2 increased as the NEFA concentration gradually increased but was lower in the high-concentration group than in the medium-concentration group and similar to the level in the low-concentration group. However, the expression of p-IRS2 in the NEFAs + TAK-242 group was lower than that in the 2.4 mM NEFAs group (*p* < 0.05). There was no difference in p-IRS2 levels between the DMSO and blank control groups, but it was higher in the TAK-242 group than in the blank control group. There was no difference in IRS2 levels between the groups (*p* > 0.05). The PI3K protein level showed no obvious difference between the NEFAs groups (*p* > 0.05), but after the addition of TAK-242, it was higher than that in the 2.4 mM NEFAs group (*p* < 0.05). The expression levels of p-AKT in the low- and medium-concentration groups were higher than those in the blank control group, but p-AKT expression in the high-concentration group was significantly lower than that in the medium- and low-concentration groups (*p* < 0.05). After the addition of TAK-242, the p-AKT level was obviously higher in the NEFAs + TAK-242 groups than in the high-concentration group (*p* < 0.05), and p-AKT levels in the TAK-242 and DMSO groups were higher than those in the blank control group (*p* < 0.05), but there was no difference in p-AKT level between the TAK-242 and DMSO groups (*p* > 0.05). The protein level of AKT did not significantly differ between the NEFAs groups (*p* > 0.05) and was higher in the NEFAs + TAK-242 group than in the NEFAs group (*p* < 0.05). The protein level of p-GSK-3β increased as the NEFAs concentration increased and was highest in the medium-concentration group but was lower in the high-concentration group than in the medium-concentration group (*p* < 0.05) and changed in a dose-dependent manner. There was no significant difference in p-GSK-3β protein levels between the other groups (*p* > 0.05). The protein levels of GSK-3β in the low- and medium-concentration groups were not significantly different from those in the blank control group (*p* > 0.05), but the protein level in the high-concentration group was lower than that in the blank control group and NEFAs + TAK-242 group (*p* < 0.05). The protein expression of GLUT4 decreased with increasing NEFAs concentrations. After the addition of an inhibitor, GLUT4 protein expression was higher in the NEFAs + TAK-242 group than in the high-concentration group (*p* < 0.05) and that in the TAK-242 and DMSO groups did not significantly differ from that in the blank control group (*p* > 0.05; Figure 8).

### NEFAs Affect the Expression of PEPCK and G6-Pase Through TLR4 at the MRNA Level

**Figure 9:**
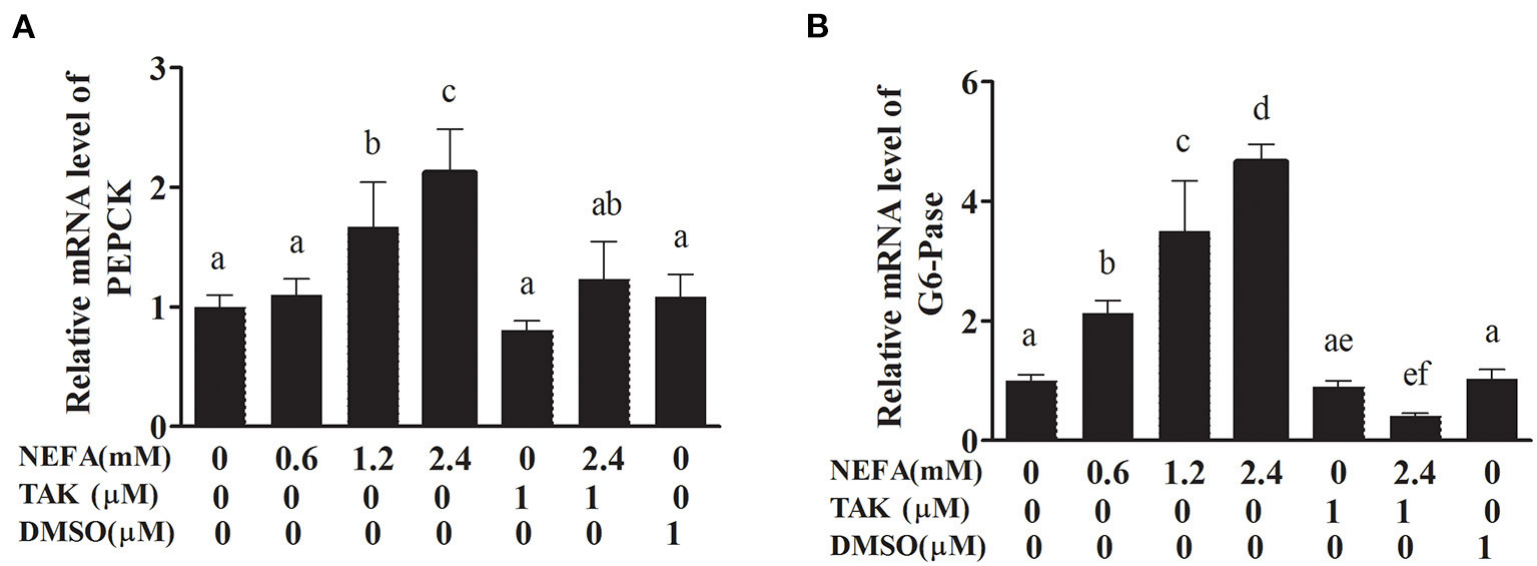
Effects of NEFAs and TAK-242 on the mRNA expression levels of PEPCK and G6-Pase. Hepatocytes were treated with NEFAs (2.4 mM), TAK-242 (1 μM), and DMSO (1 μM) for 9 h. **(A)** Relative mRNA level of PEPCK. **(B)** Relative mRNA level of G6-Pase. Values are the means ± SEs (*n* = 6), and different letters indicate statistically significant differences; *p* < 0.05.

**Figure 10:**
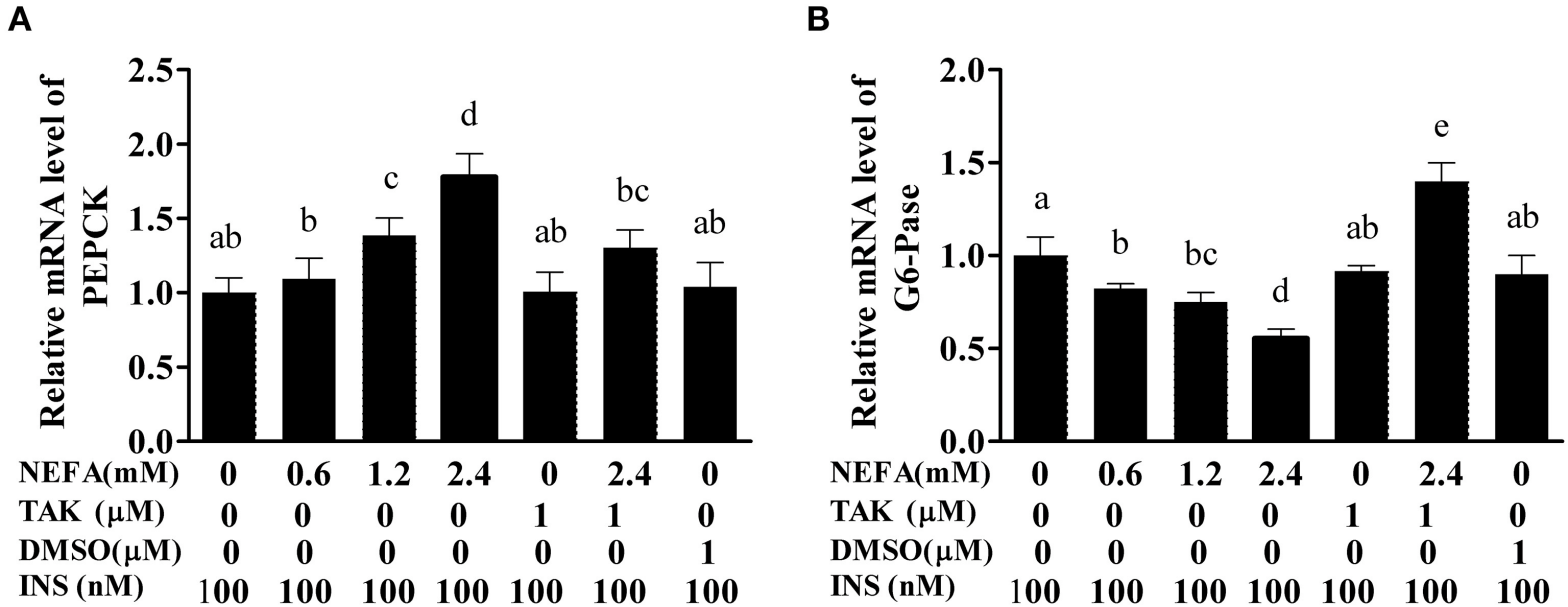
Effects of NEFAs, TAK-242, and INS on the mRNA expression levels of PEPCK and G6-Pase. Hepatocytes were treated with NEFAs (2.4 mM), TAK-242 (1 μM), and DMSO (1 μM) for 9 h and insulin (INS, 100 nM) for 1 h. **(A)** Relative mRNA level of PEPCK. **(B)** Relative mRNA level of G6-Pase. Values are the means ± SEs (*n* = 6), and different letters indicate statistically significant differences; *p* < 0.05.

PEPCK and G6-Pase are key enzymes in the gluconeogenesis process. The mRNA levels of PEPCK in the high- and medium-concentration groups were higher than those in the blank control group (*p* < 0.05), but there was no difference in PEPCK mRNA levels between the low-concentration group and blank control group (*p* > 0.05). The PEPCK mRNA level in the NEFAs + TAK-242 group was lower than that in the high-concentration group (*p* < 0.05), and there were no differences in the PEPCK mRNA level between the TAK-242, DMSO, and blank control groups (*p* >0.05; Figure 9A). The mRNA level of G6-Pase increased with increasing NEFAs concentration and was highest in the high-concentration group (*p* < 0.05). The expression level in the NEFAs + TAK-242 group was lower than that in the high-concentration group (*p* < 0.05), and there were no differences in expression between the other groups (*p* > 0.05; Figure 9B). After the addition of insulin, the mRNA level of PEPCK increased with increasing NEFA concentration, and the PEPCK mRNA level in the NEFAs + TAK-242 group was lower than that in the high-concentration group (*p* < 0.05), which was in accordance with the levels in the group without insulin (Figure 10A). The mRNA level of G6-Pase decreased with increasing NEFA concentration; the G6-Pase mRNA level in the NEFAs + TAK-242 group was higher than that in the high-concentration group (*p* < 0.05), and there were no difference in the G6-Pase level between the TAK-242, DMSO, and blank control groups (*p* > 0.05; Figure 10B).

## Discussion

Ketosis is one of the most common metabolic diseases in perinatal dairy cows (27). Furthermore, ketosis-related diseases, including fatty liver, abomasum displacement, infectious diseases and other productive diseases (28, 29). Perinatal cows are characterized by NEB (30). The initial stage of NEB in cows is characterized by low glucose and high NEFAs levels (31). Cows under NEB conditions mobilize a large amount of fat to provide more ATP to oxidize NEFAs in the cow liver (32). However, high NEFAs concentrations in the blood of perinatal cows trigger some NEB-related metabolic disorders and diseases, such as liver lipid accumulation, ketosis, abomasum displacement and retained fetal membranes (33). Clinical data show that fatty liver in cows often manifests as insulin resistance. Cows with type I ketosis often exhibit low insulin levels, and those with type II ketosis often exhibit high insulin levels with decreased insulin sensitivity (26, 34, 35). Therefore, specific liver regulation of the insulin signaling pathway is crucial to explaining the pathogenesis of cow ketosis and fatty liver. The intake of too much energy may lead to obesity and insulin resistance. TLRs are pattern recognition receptors that can affect microbial pathogens by promoting inflammatory signaling pathways such as the NF-κB pathway in the innate immune system (36). The NF-κB pathway can promote the synthesis of inflammatory cytokines and chemokines (37). Fatty acid accumulation is one of the most important factors that affects insulin resistance (38). Insulin resistance caused by fatty liver is associated with cytokines secreted by fat cells such as adiponectin and resistin and inflammatory factors such as TNF-α and IL-6 (39). Fatty liver is characterized by long-term inflammatory pathway activation, and inflammatory diseases are associated with insulin resistance in fatty liver (40). However, the mechanism of this inflammatory pathway activation is not clear in dairy cow fatty liver. High NEFAs and BHBA levels are clinicopathological characteristics of NEB (21); however, it is unclear whether insulin resistance in perinatal cows with NEB is associated with high blood NEFAs levels.

### NEFAs Affect the NF-κB Inflammatory Signaling Pathway Through TLR4

Studies in humans and mice have shown a very close connection between inflammation and insulin resistance (41–43). Rats in which TLR4 was specifically knocked out fed a high-fat diet exhibit fatty liver, but this high-fat diet can obviously increase insulin sensitivity, reducing fat accumulation and inflammation in the blood (44). The metabolic characteristics of cows are different from those of humans and rats, and the relationship between insulin resistance and inflammation in bovines is not clear. In this study, we assumed that NEFAs can bind TLR4 on the cell membrane of hepatocytes and influence the NF-κB and insulin signaling pathways.

We found that NEFAs obviously increased the initial calf hepatocyte TLR4 protein expression level, and with increasing concentrations of NEFAs, the P65 phosphorylation level also gradually increased (Figure 1). Because the activation of NF-κB is mainly achieved through the phosphorylation of p65, we are sure that NEFAs can activate the TLR4 and NF-κB pathways in hepatocytes of cows. After activation of the NF-κB pathway, a large number of inflammatory factors, such as IL-6, IL-1β, and TNF-α, are produced. To detect whether NEFAs affect the NF-κB inflammatory pathway by binding TLR4, we added a TLR4 inhibitor group and the corresponding control group (Figure 3). Our experimental results showed that the protein expression levels of TLR4 and p-P65 in the group mixed with a TLR4 inhibitor and NEFAs were significantly lower than those in the NEFAs group, and the expression of the nuclear transcription factor p65 decreased significantly after the addition of inhibitor (Figure 4). Similarly, the mRNA expression and supernatant concentration of the proinflammatory factors IL-6 and TNF-α were significantly increased in the NEFAs group but decreased after the addition of TLR4 inhibitors (Figures 5, 6). This indicates that an inhibitor of TLR4 could inhibit not only TLR4 but also p-P65, a key factor in the inflammatory pathway of NF-κB, and it can also inhibit the expression and secretion of proinflammatory factors. Overall, these findings indicate that NEFAs can activate the NF-κB pathway through TLR4, promote the release of proinflammatory factors such as IL-6 and TNF-α, and then promote the inflammatory response.

### NEFAs Affect Insulin Pathway Through TLR4

In patients with diabetes, metabolically active cells cannot use glucose, so they heavily accumulate in the blood (45). Glucose is mainly used in targeted tissues through two pathways, the PI3K and AMPK pathways (46). The PI3K/AKT pathway promotes the expression of GLUT4 in muscle cells and adipocytes and plays an important role in glucose metabolism (47). AMPK is activated mainly through drug stimulation, such as stimulation with metformin, or through exercise, effectively promoting glucose utilization and insulin sensitivity (48). Therefore, the PI3K/AKT and AMPK pathways may be potential targets for regulating insulin resistance to glucose metabolism in type 2 diabetes and obesity. In this study, we studied the PI3K/AKT pathway. IRS1 is a key regulatory factor downstream of the insulin receptor in insulin signaling. IRS1-deficient mice showed normal glucose tolerance and insulin resistance (49). Nakamura et al. indicated that although severe hyperinsulinemia occurred in IRS1^−/−^ rats fed a high-fat diet, there was no liver lipid degeneration (50). The activation of PI3K in the PI3K pathway depends on the binding of two SH2 regions to the tyrosine-phosphorylated IRS protein (51). Knockout of the PI3K regulatory subunits p85α and p85β increased insulin sensitivity (52, 53). Previous studies have shown that liver-specific knockout of PRSS8 reduced the phosphorylation of AKT in the liver, resulting in insulin resistance (54). GSK-3β is a key enzyme that regulates insulin signaling through glycogen synthase (GS) (55). GSK-3β can be activated after phosphorylation of AKT, and AKT/GSK-3β signaling plays an important role in insulin resistance (56).

In this study, we first examined whether NEFAs could affect the insulin pathway in primary calf hepatocytes. High concentrations of NEFAs increased the protein expression of p-IRS1 (Ser307), p-IRS2, IRS2, and GLUT4 but decreased the expression of PI3K, p-AKT, and p-GSK (Figure 2). Therefore, we determined that high concentrations of NEFAs can induce insulin resistance in primary calf hepatocytes. To determine whether NEFAs affect the insulin pathway through TLR4, we detected changes in the expression of key proteins in the insulin pathway after adding TLR4 inhibitors and prepared a non-insulin group as a control. After the addition of insulin, the expression levels of key proteins in the insulin pathway were greater than those in the non-insulin group; for example, the decrease in PI3K and p-AKT was more pronounced than that in the non-insulin group (Figures 7, 8). In general, the addition of TLR4 inhibitors affected the insulin pathway, indicating that NEFAs can affect the insulin pathway by binding TLR4. PEPCK and G6-Pase are two key enzymes involved in gluconeogenesis. Previous studies have shown that the activation of AKT in mouse liver can induce insulin-mediated gluconeogenesis, accompanied by downregulation of PEPCK and G6-Pase expression (57). To further verify the effect of TLR4 inhibitors on genes downstream of the insulin pathway, we detected the mRNA expression levels of PEPCK and G6-Pase in hepatocytes. In the absence of insulin, the mRNA expression levels of PEPCK and G6-Pase were increased in the NEFAs group and decreased in the inhibitor mixed group, but after the addition of insulin, G6-Pase showed the opposite change (Figures 9, 10). Because PEPCK and G6-Pase are downstream of the insulin pathway, their sensitivity to insulin is not particularly accurate, so this finding is not enough to explain its effect on the insulin pathway.

Consequently, these results indicate that NEFAs can regulate the insulin pathway through the TLR4/PI3K/AKT metabolic axis.

## Data Availability Statement

The original contributions presented in the study are included in the article/supplementary materials, further inquiries can be directed to the corresponding author.

## Ethics Statement

The animal study was reviewed and approved by Inner Mongolia University for Nationalities. Written informed consent was obtained from the owners for the participation of their animals in this study.

## Author Contributions

QD designed and performed a part of the experiment and wrote the first manuscript. All authors contributed to the article and approved the submitted version.

## Funding

This work was supported by the National Natural Science Foundation of China (Beijing, China; Grant Nos. 31602121 and 31760752), Young Scientific and Technological Talents in Inner Mongolia (Inner Mongolia, China, Grant No. NJYT-20-B30), the Natural Science Foundation of Inner Mongolia (Inner Mongolia, China, Grant No. 2021LHMS03009), Inner Mongolia Beef Diseases Prevention and Control Engineering Technology Research Center (Grant No. MDK2019023), and Dr start-up fund in Inner Mongolia University for Nationalities (BS476).

## Conflict of Interest

The authors declare that the research was conducted in the absence of any commercial or financial relationships that could be construed as a potential conflict of interest.

## Publisher's Note

All claims expressed in this article are solely those of the authors and do not necessarily represent those of their affiliated organizations, or those of the publisher, the editors and the reviewers. Any product that may be evaluated in this article, or claim that may be made by its manufacturer, is not guaranteed or endorsed by the publisher.
